# The Economic and Fiscal Impact of Influenza Vaccination for Health Care Workers in Italy

**DOI:** 10.3390/vaccines10101707

**Published:** 2022-10-12

**Authors:** Giovanna Elisa Calabrò, Filippo Rumi, Elettra Fallani, Roberto Ricciardi, Americo Cicchetti

**Affiliations:** 1 Section of Hygiene, Department of Life Sciences and Public Health, Università Cattolica del Sacro Cuore, L.go F. Vito 1, 00168 Rome, Italy; 2 VIHTALI (Value in Health Technology and Academy for Leadership & Innovation), Spin-Off of Università Cattolica del Sacro Cuore, 00168 Rome, Italy; 3 Graduate School of Health Economics and Management (ALTEMS), Università Cattolica del Sacro Cuore, 00168 Rome, Italy; 4 Department of Life Sciences, University of Siena, 53100 Siena, Italy; 5 Seqirus S.r.l., 53035 Monteriggioni, Italy

**Keywords:** fiscal impact, economic burden, social cost, productivity loss, influenza vaccination, health care workers, value, public health

## Abstract

Influenza has a significant impact on the health care system and also on production and economic systems. Vaccinated health care workers (HCWs) are more likely to have improved productivity compared to unvaccinated workers. The study aim was to estimate the economic and fiscal impact of an influenza vaccination program for HCWs in Italy. We performed a cost analysis aimed to estimate the indirect costs (productivity losses due to working days lost) and the increase in tax revenues derived from the increase in vaccination coverage among HCWs. Assuming an incremental increase in vaccination coverage of 10% per year over a period of 5 years, total savings could be obtained in terms of a reduction in productivity losses equal to −€4,475,497.16 and an increase in tax revenues of €327,158.84. This revenue could be used to finance other health interventions. Our results are fundamental in view of the sustainability of health systems and of a value-based allocation of health resources. Therefore, a complete social perspective, including the fiscal impact of flu vaccination, should be adopted to assess the economic value of influenza vaccines. Currently, health policies based on the whole value of flu vaccination are needed.

## 1. Introduction

Influenza (or the flu) is an acute infectious viral respiratory illness that leads to seasonal outbreaks and, rarely, pandemics [1]. The global estimates of the World Health Organization (WHO) report 290,000–650,000 influenza-associated respiratory deaths every year [2]. Moreover, influenza is reputed among the infectious diseases to have the highest burden on population health in Europe [3], where it causes 4–50 million symptomatic cases annually, approximately 15,000–70,000 deaths’ and 150,000 influenza-related hospital admissions [4]. Therefore, the significant morbidity and mortality related to seasonal influenza, especially in vulnerable individuals such as children and elderly individuals, cause an increased economic burden on both the health system and society annually [4].

The WHO divided vulnerable individuals who have a greater risk of contracting influenza, transmitting it, and developing complications, into five risk categories, namely, children under 5 years of age, pregnant women, people over 65, chronic patients, and health care workers (HCWs) [5]. However, despite WHO recommendations, influenza vaccination policies are consolidated only in high-income countries and in a few low- and middle-income countries [6], and in some risk categories, such as HCWs, suboptimal vaccination coverage is reported worldwide [7].

HCWs are at an increased risk of exposure to influenza, posing a potential threat to their health and to patient safety. A systematic review and meta-analysis of the flu incidence among medical personnel and other healthy adults by Kuster et al. [8] (58,245 participants in total; influenza seasons 1957–2009) suggested that HCWs have a higher risk of symptomatic influenza infections (up to 2.5 times) compared to the population of healthy adults working in settings other than health care facilities. According to the estimates of Kuster et al. up to 22% of HCWs (especially those not vaccinated against influenza) can have influenza every epidemic season [8]. Therefore, flu vaccination is considered as a key preventive intervention of control activities for the prevention of healthcare-associated influenza (HAI) transmission and to reduce patient morbidity and mortality, to increase patient safety, and to reduce work absenteeism among HCWs [9].

Furthermore, several studies reported data on presenteeism associated with influenza-like illness (ILI) among HCWs [9,10,11]. Results of these studies show that a large group of physicians (even >75% [11]) admit to carrying out their work despite having flu-like symptoms (the so-called presenteeism). For example, according to the results of an American survey (involving 1914 HCWs; influenza season 2014–2015), 41.4% of the respondents reported being present at work with influenza-like symptoms (a median of 3 days), while pharmacists and physicians were the ones who most commonly reported being present at work when sick (67.2% and 63.2%, respectively) [12]. In this way, HCWs can promote influenza virus transmission, putting patients at risk [11].

Globally, it is estimated that the HCWs influenza vaccination rates range from 2–44% and the recommended optimal influenza vaccination coverage rate for medical personnel to protect patients is about of 90% [11].

Nevertheless, suboptimal vaccination coverage is also reported in Europe [13]. Although several European countries have specific vaccination programs for HCWs, a significant proportion of them remain susceptible to influenza because they are unvaccinated [14].

The most recent European report about the seasonal influenza vaccination coverage rate detected suboptimal adherence among HCWs for the 2015–2016, 2016–2017, and 2017–2018 seasons, ranging from 63.2% in Belgium to 15.6% in Italy [15]. Nonetheless, a considerable intensification of flu vaccine uptake was observed in Italy during the 2020–2021 influenza season, at least partly as a consequence of the SARS-CoV-2 pandemic [16,17]. The maintenance of this positive trend is desirable, since HCWs model the health behavior to follow, advising patients and educating by example, and they have the responsibility to protect themselves to protect their vulnerable patients [18].

Therefore, vaccination is the most effective shield against influenza [19] both because it greatly increases the probability of not contracting the disease and because it lessens the severity of flu symptoms, which are generally, not followed by further complications. Furthermore, influenza vaccination represents an important protective measure not only for individuals, but also for the community [4]. It reduces the probability of complications and consequently the impact in terms of health care burden (hospitalizations, outpatient visits, and medications) [20], and the impact on children’s school absences and missed working days, either due to secondary illness in a caregiver or the need to care for a sick child [4], and on society, in terms of productivity losses and workers’ absenteeism [13].

Indeed, vaccine-preventable diseases (VPDs), such as influenza, have a significant impact not only on the health and social care system, but also on the production and economic systems. By decreasing the morbidity and mortality of VPDs, vaccinated workers are more likely to have improved productivity, work for more time, and remain active and prolific for longer in the job market than unvaccinated workers [21]. Moreover, flu immunization is recommended during seasonal epidemics to ensure proper functionality of health services and prevent presenteeism and absenteeism [11,22].

An Italian study conducted in a large hospital in Rome during the 2017–2018 influenza season estimated a distinctly lower loss of productivity per capita in vaccinated HCWs compared to unvaccinated HCWs (respectively, €297.06 and €517.22), leading to a cumulative difference of 120.07 € for each undisposed day by applying the so-called human capital approach. The same calculations have been performed using an alternative method, the friction cost model, achieving the same results, even if in a less pronounced way [23].

The relevance of the economic burden of respiratory diseases in HCWs has been further highlighted by a prospective Swiss surveillance study [24]. The study, performed during two consecutive flu seasons (2015–2016 and 2016–2017), reported that nearly 90% of the health care professionals analyzed had manifested at least one influenza symptom, and 28% had missed one or more working days. In addition, 68% of the participants had gone to work despite the presence of influenza symptoms, introducing the problem of presenteeism with respiratory diseases, which represents a threat for colleagues and patients [24]. In contrast, a systematic review and meta-analysis found a non-significant influence of flu vaccination on ILI incidence among HCWs, although a strong benefit was observed when the outcome became more specific and analyzed only the laboratory-confirmed influenza case rate [25].

Therefore, given the importance of appropriately allocating the available resources of the health care system, the development of new health economic models is needed to guide policy-makers in a value-based evaluation of immunization strategies that take into account the whole value of vaccination [26]. The economic impact of vaccinations should incorporate health and non-health benefits of vaccination in both vaccinated and unvaccinated populations, thus allowing for estimation of the societal value of vaccination [27]. When assessing the economic value of vaccines, decision-makers should adopt a full societal perspective that also considers the fiscal impact of an infectious disease [28]. The fiscal health model assumes that a higher productivity of HCWs translates into increased individual income, resulting in additional government tax revenues available to reinvest in health care services and the workforce. If an illness decreases the individual productivity, all the systems are negatively affected [28].

Ruggeri et al. [28] assessed the fiscal impact of influenza, pneumococcus, and herpes zoster vaccines in Italy through the human capital approach, focusing on the general population aged 30 to 65. The study concluded that a flu vaccination program able to avert 200,000 influenza infections would increase the annual productivity by approximately €111 million and the fiscal revenue by approximately €18 million.

Therefore, the main objective of the present study was to use the theoretical framework developed by Ruggeri et al. [28] to estimate the economic and fiscal impact of an influenza vaccination program for HCWs in Italy.

## 2. Materials and Methods

We assessed the economic impact of an influenza vaccination program among HCWs in Italy by considering direct health care costs, productivity losses, and the fiscal impact. Specifically, the analysis applied the theoretical framework proposed by Ruggeri et al. in 2019 [28]. According to this framework, the accumulation of human capital and the increase in population health are key factors of economic growth in a country. Therefore, the investment in new health technologies capable of increasing population health correlates with the increase in workers’ productivity. Increasing productivity increases incomes, therefore consumption, and tax revenues also increase, which in turn could be used by governments to invest in health [28].

We conducted the analysis in two stages. First, we estimated the number of HCWs exposed to the flu. Second, we performed a cost analysis aimed to estimate the indirect costs (productivity losses due to working days lost) and the increase in tax revenues deriving from the increase in vaccination coverage among HCWs. For the estimation of social costs due to productivity losses, and therefore, to estimate the influenza fiscal impact, we used the human capital approach [28]. According to this approach, the individual “produces” in proportion to the income received, and the salary corresponds to the effective contribution of the worker to the productive activity [28].

Epidemiological data were extrapolated from the literature. Finally, the analysis took into account the vaccination coverage among HCWs that ranged between 30% and 70%. The results were reported by the number of HCWs exposed to the flu, the indirect costs avoided, and the total increase in tax revenue.

### 2.1. Fiscal Impact Estimation

As reported by Ruggeri et al. [28], we defined fiscal impact as the decrease in tax revenue resulting from the reduction in individual income due to a specific condition/disease. Tax revenues are typically derived from individual income, which is correlated with productivity. Productivity in turn strongly depends on people’s health. Therefore, the sustainability of health care systems may depend on their ability to ensure high levels of productivity through maintaining or improving health.

The purpose of our evaluation was to test the analytical framework developed by Ruggeri et al. 2019 [28] to estimate the global impact (direct, indirect, and fiscal) of a flu vaccination program for HCWs in Italy. Our assessment was consistent with the background of the theoretical framework and assumed that the accumulation of human capital and the increase in population health are the key drivers of economic growth and the results of an endogenous process [28]. Therefore, according to this perspective, governments should invest in new health technologies to increase population health, thus improving production. Increasing productivity increases incomes, therefore consumption, and tax revenues, which in turn can be used to increase investment in health. This process can be simplified with the following cause and effect formula:

$$\overset{+H\;\overset{yields\;}{\to }+y\;\overset{yields\;}{\to }+W\;\overset{yields\;}{\to }+T\;\overset{yields\;}{\to }+G}{\leftarrow },$$

where *H* represents the number of healthy individuals, *y* represents employer productivity, *W* represents employee income, *T* represents total tax revenue, and *G* represents public health expenditure [28].

### 2.2. Cost Estimation

To determine the fiscal impact related to the increase in vaccination coverage among HCWs, a simulation was conducted to identify avoided productivity losses, tax revenues, and social costs associated with the flu syndrome. The model considered an influenza attack rate in an unvaccinated cohort of 4.4% [29] and vaccination coverage in the HCW group of 30% (assumption). These two parameters were useful in defining the HCWs who could have contracted the influenza virus.

The model also assumed that HCWs receiving the vaccine do not contract the flu. Then, an average of the total number of working weeks and weekly working hours for Italian HCWs was estimated. Furthermore, an average number of 48 weeks of work and a total of 44 h per week were assumed. The model broke down the hourly cost for each professional of the National Health Service (NHS) by identifying the fixed part on the gross taxable amount (83%) and the variable part (17%). The cost of one hour of work for an HCW, considering both specialists and other HCWs (such as nurses, midwives, pharmacists) in Italy, was €35.04. Therefore, a fixed part of €29.08 and a variable part of €5.96 were considered in the simulation. On the basis of this information, the model calculated the total weekly average gross taxable amount of €1541.76 and the annual average gross taxable amount of €74,004.48. Considering an average duration of flu symptoms of two days (conservative assumption based on Italian National Institute of Health data [30]), the productivity losses expressed in the number of hours lost due to flu syndrome (16 working hours) were calculated. Thus, flu syndrome could potentially generate an impact on the total annual taxable amount of €73,909.17 (compared to €74,004.48, which is the total annual taxable amount). The model calculated an average annual tax revenue of €24,991.93 for HCWs compared to €24,950.94 among those who contracted the influenza. Thus, the tax impact for HCWs was estimated to be €40.98 for flu syndrome episodes. Table 1 shows the estimates described above.

### 2.3. Eligible Population

The eligible population was extrapolated from the latest available data referring to 2021 and provided by the Italian National Institute of Statistics [31].

Table 2 reports the types of HCWs, the incidence on 10,000 inhabitants, and an average annual salary. The total number of HCWs in Italy was 753,658 in 2021. The model considered the total number of HCWs potentially exposed to flu. Assuming a vaccination coverage of 30% and an influenza attack rate for an unvaccinated cohort equal to 4.4% [29], the analysis considered a total of 23,213 flu cases among the HCWs using the following formula:

Total number of HCWs ∗ (1−vaccination coverage among HCWs) ∗ attack rate among unvaccinated cohort

### 2.4. Sensitivity Analysis

A sensitivity analysis was provided by varying the vaccination coverage value among HCWs. This specific analysis allowed us to understand the implications from the point of view of (indirect) social costs and fiscal impact. In fact, vaccination coverage is potentially able to reduce the absenteeism of HCWs. This implies a reduction in productivity losses and at the same time an increase in tax revenues. The results of the model were presented starting from a vaccination coverage of 30% (base case) and assuming an incremental trend of 10% up to 70%.

## 3. Results

On the basis of the estimate of the fiscal impact, an incremental increase in vaccination coverage among HCWs identified in the Italian setting was assumed in our model. Therefore, the model provided a simulation, starting at 30% vaccination coverage and increasing in increments of 10% to estimate the impact resulting from the increase in terms of additional tax revenue and indirect costs avoided. The objective of the simulation is, therefore, to estimate the reduction of indirect costs derived from the lower number of professionals affected by the flu (expressed in lost working days) and from the increase in tax revenues made from the reduction of working days lost due to flu symptoms (fiscal impact).

**Figure 1:**
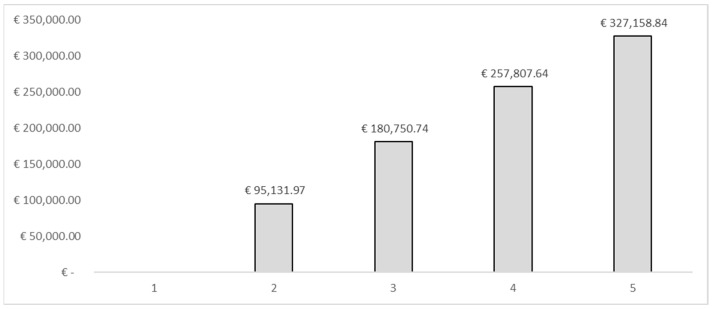
Simulation—Fiscal impact (cumulated).

**Figure 2:**
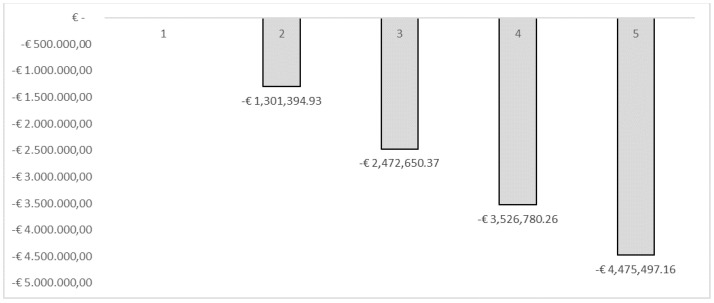
Simulation—Indirect costs (cumulated).

**Figure 3:**
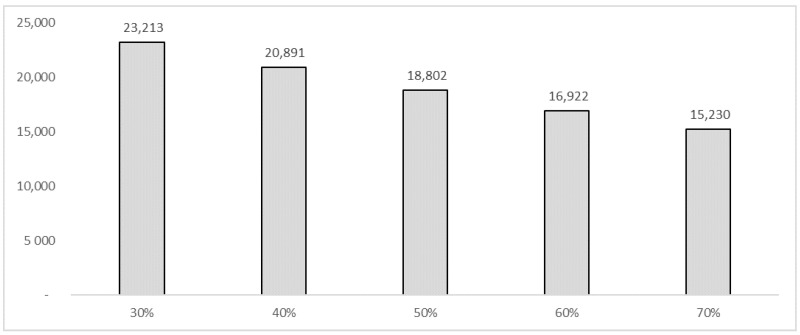
Simulation—Number of influenza cases among HCWs.

Table 3 shows the results of the analysis. We estimated that the 10% (from 30% to 40%) increase in influenza vaccination would be able to bring benefits (savings) in terms of social costs equal to −€1,301,394.93, taking into account the cases of influenza avoided being equal to 2321. These results were estimated using the human capital approach. With regard to the fiscal impact, the same percentage of variation in vaccination coverage would be able to increase tax revenues by €95,131.97. This revenue, consistent with the fiscal impact framework, could be used to finance interventions in the field of public health, generating more health, which in turn could generate a population with less loss of productivity and higher tax revenues. The cumulative results assuming an incremental increase in vaccination coverage among HCWs of 10% per year over a 5-year period within the simulation conducted would lead to a total saving in terms of reduction of productivity losses expressed in the form of days of work lost due to the flu syndrome equal to −€4,475,497.16. In terms of the increase in tax revenues, the cumulative result estimated a value of €327,158.84. Figure 1, Figure 2 and Figure 3 show the results of the analysis stratified by percentage of vaccination coverage.

As seen in the analysis carried out, an increase in vaccination coverage leads to an increase in terms of tax revenues derived from the minor number of HCWs affected by the flu virus and involves a reduction in indirect costs (productivity losses) because professionals lose fewer days of work on average.

The model did not consider the direct health costs related to the acquisition and administration of the flu vaccination. In addition, within the simulation, a vaccine efficacy in the prevention of influenza cases equal to 100% was assumed. However, in light of the results obtained, it is possible to believe that an extension of the vaccination campaign among HCWs could represent a cost-saving strategy with a view to the efficiency of resource allocation. This is because direct health care costs are likely to be offset by using a broader perspective. Therefore, we believe that the savings in terms of indirect costs and the greater benefits derived from the increase in tax revenues can amply offset the costs of acquiring and administering influenza vaccines among the target population of our simulation.

## 4. Discussion

In our study, we implemented a theoretical framework on fiscal impact [28] to estimate the economic impact of an influenza vaccination program for HCWs in Italy by considering direct health care costs, productivity losses, and fiscal impact. According to the proposed framework [28], the increase in the productivity of vaccinated HCWs due to the absence of flu symptoms increases their incomes and therefore consumption and tax revenues, which in turn could be used by governments to implement investments in health.

In our analysis, first, we estimated the number of HCWs exposed to the flu; second, we performed a cost analysis aimed to estimate the reduction of indirect costs (productivity losses due to working days lost) and the increase in tax revenues derived from the increase in vaccination coverage among HCWs. Therefore, the analysis took into account the vaccination coverage among HCWs that ranged between 30% and 70%.

Assuming a vaccination coverage of 30% and an attack rate of influenza equal to 4.4% [29], the analysis considered a total of 23,213 HCWs exposed to seasonal influenza virus. Estimating an increase in vaccination coverage among HCWs of 10% (from 30% to 40%), savings in terms of social costs of −€1,301,394.93 could be achieved. These results were estimated using the human capital approach. The same percentage of variation in vaccination coverage would be able to increase tax revenues by €95,131.97. Furthermore, assuming an incremental increase in vaccination coverage among HCWs of 10% per year over a period of 5 years, total savings could be obtained in terms of a reduction in productivity losses equal to −€4,475,497.16 and an increase in tax revenues of €327,158.84. This revenue could be used to finance interventions in the public health field, generating more health, which in turn could generate a population with less loss of productivity and consequently higher tax revenues.

Therefore, an increase in vaccination coverage among HCWs leads to an increase in terms of tax revenues derived from the minor number of professionals affected by the influenza and at the same time involves a reduction in indirect costs (productivity losses) because HCWs lose fewer days of work on average.

The optimization of the limited resources and the economic and financial sustainability of health systems have become central topics in the discussion of the health sector over the last few years [26]. In this context, flu vaccination in children, adults, and elderly individuals results in a reduction in hospitalizations, ambulatory care visits, and medical interventions, which leads to substantial savings in health care costs each year in Europe and worldwide [33]. Furthermore, this vaccination among HCWs is associated with a substantial decrease in mortality for elderly patients; therefore, the cost of not vaccinating HCWs can also be substantial in terms of missed benefits [33]. Nevertheless, influenza vaccination is associated with a reduction in indirect costs in terms of lost productivity and days of work lost due to illness [4,8] and reduces the potential fiscal impact caused by the disease [28]. Therefore, flu vaccination represents an exceptional opportunity to keep people healthy and can contribute to the sustainability of health care systems by evading unnecessary use of financial and human resources and freeing resources for other health interventions [33]. These principles are perfectly in line with what is currently proposed within value-based health care (VBHC). Indeed, recently, the Expert Panel on Effective Ways of Investing in Health (EXPH) of the European Commission (EC) proposed a value-based long-term strategy that provides a reallocation of resources from low- to high-value care to free resources for reinvestment in health [34]. In particular, the EXPH proposed VBHC as a comprehensive concept built on four value pillars: appropriate care to achieve patients’ personal goals (*personal value*), achievement of best possible outcomes with available resources (*technical value*), equitable resource distribution across all patient groups (*allocative value*), and contribution of health care to social participation and connectedness (*societal value*) [34]. This approach has also been applied to the vaccination field, emphasizing the importance of evaluating and communicating the whole value of vaccines and vaccination [26,35]. Understanding value in health should be shared by all stakeholders and geared toward the goal of maximizing social wellbeing. In fact, in recent years, we have moved from the concept of value-based health care to the concept of a value-based health system, as it is the whole health system that contributes to the wellbeing of society due to prevention and health promotion [36]. Nonetheless, despite the effectiveness and cost-effectiveness of prevention interventions, investment in prevention remains low in many countries [37]. Moreover, this is reflected, for example, with vaccination coverage still not optimal for the main VPDs, including influenza, in Europe and elsewhere. For this reason, health policies and immunization strategies based on the whole value of vaccination are needed. To achieve these ambitious goals, strengthening the generation of evidence and data is necessary to guarantee an evidence-based decision-making process in the context of influenza vaccination. Furthermore, new evidence-based assessment frameworks and tools capable of recognizing the whole value of flu vaccines and vaccination are indispensable [26]. This challenge also evidently relates to the need to better assess the impact of flu vaccination at the societal level. The economic impact of influenza vaccination should incorporate the health and non-health benefits of vaccination, both in the vaccinated and in the unvaccinated population, thus allowing estimation of its societal value [27]. Therefore, the development of health economic models capable of capturing not only the cost-benefit of vaccination, but also the value of health itself is needed [38].

Health care decision-makers and policy-makers should be aware of the limitations of traditional economic assessments for evaluating vaccine value [39]. Future economic evaluations should pay more attention to the effect of vaccination on complication prevention, the generation of health benefits for HCWs, and benefits for the community beyond individual protection; in addition, guidelines for the economic assessment of the whole value of vaccinations are needed, and economic analysis must be conducted taking into account the societal perspective as well as that of the health system to underline and prove the whole value of vaccines [39]. From the societal perspective, influenza vaccination represents an important protection measure not only for individuals but also for the community, and it is cost-effective for children, pregnant and postpartum women, high-risk groups, and healthy working-age adults [40]. Therefore, a complete social perspective should be adopted to assess the economic value of vaccines. Traditional methods to estimate illness costs from a social perspective can also be improved by considering the fiscal impact, which explains the decline in disease fiscal revenues. The potential reduction of the fiscal impact should be included in the evaluation of vaccines and vaccinations, adding a new dimension to this valorization [28].

In our analysis, we have shown that increasing influenza vaccination coverage among HCWs can reduce productivity losses and increase fiscal revenues that could be used to finance other health interventions, such as the implementation of immunization strategies against influenza among HCWs.

Influenza vaccination among HCWs has been an important topic of study in Italy in recent years, particularly with the aim of evaluating the effectiveness of vaccination campaigns and increasing vaccination coverage [41,42,43,44,45,46]. Increasing vaccination coverage among HCWs is not always guaranteed and is often a difficult goal to achieve. In fact, it depends on several variables related to the availability and delivery of the flu vaccine, the presence of adequate and expert human resources, health education, and the promotion of well-structured communication campaigns [44]. A fundamental element to increase the adherence to influenza vaccination by health professionals appears to be linked to the effectiveness of vaccination campaigns. In particular, the use of different communication approaches such as posting explanatory leaflets and posters in each hospital ward, distributing information material, creating promotional spaces—through the use of social media and conveying correct communication through websites dedicated to vaccination [44]—and using innovative methods such as forum theatre [45], have led to an increase in vaccination coverage against influenza among Italian HCWs. Other effective modalities were the organization of dedicated courses for HCWs, the active invitation to vaccination through e-mail, the on-site vaccination intervention, and the organization of dedicated units for the influenza vaccination of HCWs in the hospital setting [41,42,43,46]. However, from the literature data it is clear that the best strategy to promote flu vaccination among HCWs should include multiple approaches in order to obtain an increasing coverage trend in all health care settings [46].

On the other hand, the most recent literature criticizes the policy of mandatory flu vaccination among HCWs pointing to the lack of reliable empirical evidence on the real benefits for patients [11]. Mandatory vaccinations for HCWs have been in place in few European countries and for specific VPDs with varying success [47]. In contrast, mandatory influenza vaccination policies were widely adopted by health care facilities in the United States the past decade with uptake rates of >90% among HCWs working in hospitals [48]. In Italy, the National Vaccination Plan (PNPV 2017–2019) strongly recommends influenza vaccination of HCWs [49]. This condition allows each HCW to choose whether to get vaccinated or not. In addition, the individual Italian Regions have adopted different measures on vaccinations, especially in the workplace [50]. Given the available and multi-year evidence in the United States, in terms of high uptake rates on one hand, and the fact that voluntary flu vaccination programs have failed to achieve high coverage rates, the implementation of mandatory flu vaccination policies could be justified in order to prevent the transmission of influenza through HCWs [50]. Therefore, flu vaccination policies for HCWs will need to be reviewed and health care professionals will need to be prepared to address these issues in the next years.

In our analysis, we have shown that increasing influenza vaccination coverage among HCWs can lead to significant savings. These results are fundamental in view of the sustainability of health systems and of a value-based allocation of health resources.

Furthermore, our findings underscore the importance of investing in and using more effective flu vaccines. In fact, only by using more effective vaccines it is possible to obtain a greater impact of vaccination in terms of economic savings for the health system and for society. In light of the results obtained, it is clear that promoting influenza vaccination strategies among HCWs is a priority to be implemented to increase vaccination coverage in this target population, guarantee health benefits for the health professionals themselves and their patients, and contribute to the sustainability of health systems through a value allocation of health resources.

Our study has some limitations, the main one being that most of the data were determined only on the basis of scientific literature and assumptions. However, to overcome the lack of robustness associated with the values considered in the analysis, a sensitivity analysis was conducted. Furthermore, our results are conservative, as, for example, we only considered two working days lost due to the flu, knowing that it could be even more (4 to 6 days according to the literature data [51]). Furthermore, the impact of presenteeism, which is difficult to estimate in economic terms, has not been considered. The analysis also considered an average salary among HCWs without considering the differences between the various professional figures. The use of a weighted average and the retrieval of more specific information on professionals most exposed to contagion with the flu virus could provide more detailed information on indirect costs and the resulting fiscal impact.

Moreover, the model did not consider the direct health costs related to the acquisition and administration of the flu vaccination, but our goal was not to assess the economic and fiscal impact of specific flu vaccines. We wanted to determine the fiscal impact related to the increase in flu vaccination coverage among HCWs regardless of the vaccine used. In light of the results obtained, it is possible to believe that an extension of the vaccination campaign among HCWs could represent a cost-saving strategy because direct health care costs are likely to be offset by using a broader perspective. In fact, the savings in terms of indirect costs and the greater benefits derived from the increase in tax revenues can amply offset the costs of acquiring and administering the influenza vaccines.

However, in the future, it will also be necessary to evaluate the fiscal impact of influenza vaccination strategies with specific vaccines because the results of the model can be significantly influenced by their effectiveness.

Nevertheless, our economic model, which is the second to have applied the fiscal impact framework to influenza vaccination, may broaden the knowledge on the impact of influenza in the Italian setting and, therefore, support decision-makers in defining vaccination health policies based on a broader value of available flu vaccines. In fact, new evidence of the value of the different available flu vaccines is crucial to promote their appropriate use and to support the implementation of value-based and evidence-based immunization strategies [20].

Among health technologies, vaccines are one of the most successful in the contemporary era. Technological innovation can lead to high costs and severe financial pressure on health systems. However, health systems cannot give up on this innovation and must consider all stakeholders: citizens and patients should be guaranteed quick and equitable access to more effective health technologies; research and development efforts should be encouraged when oriented toward the production of high-value technologies; decision and policy-makers should support innovation by using evidence-based tools for their assessment; and health systems should promote technological innovation while ensuring their sustainability [52].

Furthermore, in light of the important scientific progress of recent years and the countless health needs of the population, it will be necessary to focus on value-based, but also personalized, prevention. In fact, the principles of personalized medicine have already been applied in the vaccinomics and adversomics fields to better understand interindividual variations in vaccine-induced immune responses and vaccine-related adverse events [53]. New knowledge in these areas will also help determine the “right” type or dose of vaccine for the “right” person; therefore, these aspects will also need to be included in the evaluation of a broader value of vaccines.

## 5. Conclusions

Influenza has a significant impact not only on the health care system but also on production and economic systems. Vaccinated workers are more likely to have improved productivity, work for more time, and remain active and prolific for longer in the job market than unvaccinated workers. This also applies to health care professionals. In fact, flu vaccination is recommended during seasonal epidemics to ensure proper functionality of health services and prevent absenteeism of HCWs.

Influenza prevention in children, adults, elderly individuals, risk groups, and HCWs through vaccination represents an exclusive chance to keep people healthy and to reduce the economic impact of influenza on health systems and society. In particular, vaccination of HCWs and other employees reduces the loss of productivity and the fiscal impact of the illness. Increasing productivity increases incomes, consumption, and tax revenues, which in turn can be used to increase investments in health. Hence, flu vaccination among HCWs can contribute to the sustainability of health care systems by avoiding the unnecessary use of health resources and freeing resources for other health interventions. Improving the uptake of flu immunization programs is critical for health care systems looking for more efficient and value-based health care resource use.

Therefore, the widespread promotion of influenza vaccination and the implementation of health policies aimed at increasing vaccination coverage in all target populations are key factors for the long-term sustainability of health systems around the world.

## Figures and Tables

**Table 1:** Fiscal impact and indirect cost estimation.

Variable	Value	Source
Total working hours/HCWs	2112	Italian National Institute of Statistics, 2022 [31]
Total working hours/week	44	Assumption
Hourly cost	€35.04	Calculated
Taxable Hourly Fixed Part	€29.08	Calculated
Taxable Hourly Variable Part	€5.96	Calculated
Total Weekly Taxable Amount	€1541.76	Calculated
Total Annual Taxable Amount	€74,004.48	Calculated
Number of working days lost (flu syndrome average duration)	2	Italian National Institute of Health, 2022 [30]
Number of working hours lost due to the flu syndrome	16	Calculated
Working hours considering an episode of flu/HCWs	2096	Calculated
Impact of influenza complications on the total potential/man hours	2112	Calculated
% people with flu complications	0%	Assumption
Impact of flu and complications among the HCWs total annual taxable income	€73,909.17	Calculated
Flu impact on the patient’s total annual taxable income	€73,909.17	Calculated
Tax on personal income (Italy)		
€15,000	23.00%	Italian budget law 2021 [32]
€28,000	27.00%	Italian budget law 2021 [32]
€55,000	38.00%	Italian budget law 2021 [32]
€75,000	41.00%	Italian budget law 2021 [32]
€80,000	43.00%	Italian budget law 2021 [32]
Annual income (no flu)	€24,991.93	Calculated
HCW annual revenue	€24,950.94	Calculated
HCW tax impact	€40.98	Calculated
Annual social costs per HCWs	€560.64	Calculated

**Table 2:** Eligible population considered for the model (data referring to the Italian population for 2021 [31]).

	Type of HCWs	Type of Data Health Personnel	Health Personnel per 10,000 Inhabitants	Average Annual Salary
A	Anesthetists	12,226	2.06	€76,900
	Cardiologists	13,706	2.31	€98,000
	Surgeons	8098	1.36	€125,000
	Gastroenterologists	3543	0.6	€55,000
	Geriatricians	4178	0.7	€64,900
	Neurologists	6658	1.12	€64,900
	Oncologists	4633	0.78	€86,400
	Orthopedists	9277	1.56	€95,000
	Otolaryngologists	4336	0.73	€94,700
	Urologists	4053	0.68	€86,400
B	Other Medical Specialists	109,497	18.42	€80,146
C	Pediatricians	16,569	2.79	€64,900
	Free Choice Pediatricians (FCPs)	7285	1.23	€64,900
	Pediatricians (Excluding FCPs)	9284	1.56	€64,900
(A + B + C)	Medical Specialists	187,490	31.54	€84,533
D	General Practitioners	50,354	8.47	€105,000
	General Practitioners (GPs)	41,707	7.02	€80,934
	Other doctors (Excluding GPs)	8647	1.45	€77,545
(A + B + C) + D	Total Doctors (General Practitioners and Specialists)	237,844	40.01	€79,279
E	Dentists	51,678	8.69	€77,000
	Midwives	17,239	2.9	€33,600
	Nurses	373,064	62.76	€26,400
	Pharmacists	73,833	12	€26,500
(A + B + C) + D + E	Total HCWs	753,658	128	€74,471

**Table 3:** Main results of the fiscal impact of influenza vaccination for HCWs in Italy.

Year	Vaccine Coverage	Number of HCWs Exposed to Influenza	Fiscal Impact	Indirect Costs	Total	Increase in Tax Revenues (Cumulated)	Reduction in Loss of Productivities (Cumulated)
1	30%	23,213	−€951,319.69	€13,013,949.29	€13,965,268.98	€—*	€—*
2	40%	20,891	−€856,187.72	€11,712,554.36	€12,568,742.09	€95,131.97	−€1,301,394.93
3	50%	18,802	−€770,568.95	€10,541,298.93	€11,311,867.88	€180,750.74	−€2,472,650.37
4	60%	16,922	−€693,512.06	€9,487,169.03	€10,180,681.09	€257,807.64	−€3,526,780.26
5	70%	15,230	−€624,160.85	€8,538,452.13	€9,162,612.98	€327,158.84	−€4,475,497.16

* Each year, the number of HCWs potentially exposed to influenza decreases by 10% (due to increased vaccination coverage). Therefore, in the first year, the increases are 0.

## Data Availability

Not applicable.
